# Fashion

**Published:** 1892-11

**Authors:** 


### HALL'S JOURNAL OF HEALTH

TRUTH DEMANDS NO SACRIFICE; ERROR CAN MAKE NONE.

Vol. 39. NOVEMBER, 1892. No. 11.

### FASHION.

How great is the power of fashion ! Viewed as the sovereign of the social orders, she is a relentless tyrant, commanding obedience to her decrees at whatever cost or sacrifice. She invades the saloon, the boudoir, the nursery, and even the sanctuaries of worship, whilst upon the great highways, between villas and palaces, she triumphs in queenly magnificence. Yesterday, she demanded a skirt swelled to hogshead-like proportions ; to-day, it is long trailing robes that sweep the ground like the tail of a peacock ; to-morrow, we may look for some new absurdity. It is in vain that the sober head of the family demurs, ridicules and remonstrates. "Out of fashion, out of the world," his good wife and charming daughters exclaim ; and in a sense they are right, for fashion admits into her world none other than her votaries ; it is the *caste* line over which no homely apparelled venturer is allowed to pass, be she endowed with the wisdom of an Aspasia, or the wit of a de Staël. It all lies in the cut of a garment. But when the cast-off vesture is made to serve the economy of the household by descending to the housemaid, it is only the sign of a by-gone day. Then comes the reign of flounces and furbelows, or the scrimpily cut garb that hugs the figure like bark of a tree, for it will never do for mistress and housemaid to appear in the same guise, much as the latter might desire it. Even now we have the incoming of a re-established abomination. Fashion has decreed it and so must it be. The long flowing trails are to sweep our streets once more. They are even now upon us, and we must make way for them, even to the endangerment of the family health.

Doctor Holmes, in his "Professor at the Breakfast-table," makes one of his characters say: "Our landlady's daughter is a young lady of some pretensions to gentility. She wears her trains very long as the great ladies do in Europe. To be sure, their dresses are so made only to sweep the tapestried floors of chateaus and palaces, as those odious aristocrats on the other side do not go dragging through the mud in silks and satins, but, forsooth, must ride in coaches when they are in full dress. It is true that, considering various habits of the American people, also the little accidents which the best kept sidewalks are liable to, a lady who has swept a mile of them is not exactly in such a condition that one would care to be her neighbor. Why, there isn't a beast or a bird that would drag its tail through the dirt in the way these creatures do their dresses!

"Because a queen or a duchess wears long robes on great occasions, a maid-of-all-work or a factory girl thinks she must make herself a nuisance, by trailing through the street, picking up and carrying about with her—pah! That's what I call getting vulgarity into your bones and marrow. If any man can walk behind one of these women, and see what she rakes up as she goes, and not feel squeamish, he has got a tough stomach. I would not let one of them into my room without serving them as David served Saul at the cave in the wilderness—cut off his skirts, sir! cut off his skirts! . . . . Don't tell me that a true lady ever sacrifices the duty of keeping all about her sweet and clean to the wish of making a vulgar show. There are some things that no fashion has any right to touch, and cleanliness is one of these things."

Commenting upon the filthiness of this recurring absurdity, a late issue of the *London Lancet* holds the following sentiments: "Granted that the germs of disease abound in a given quarter, no ordinary means could more effectually insure their disappearance than the broom-like action of a flowing skirt." Corroborative of this statement "Good Health" presents the following view: "Any one who has the slightest knowledge of microscopy or bacteriology will readily recognize the truth of the statement that nowhere are germs, and germs of the most dangerous and miscellaneous character to be found so abundantly as in street dust. The expectorated matter of the thousand passers-by on the sidewalk or public highway contain every sort of germ which infects the human body. In the dried sputa of the multitudes who pass one another upon the street, expectorating here and there, may be found the germs of diphtheria, pneumonia, erysipelas, cancer, consumption, and other most formidable microbes, together with those of a less dangerous character, such as those giving rise to decay of the teeth, sore eyes, inflammation of the ears, and numerous other minor maladies. Horses and other animals traversing the street are constantly soiling the pavement with excreta, which contains multitudes of microscopic organisms capable of producing various forms of disease, including the eggs of tapeworms and other parasites. The smallest bit of street dust planted upon a sterilized potato, will grow in a short time to proportions really prodigious, when compared with the original particle. It is only by such studies that one can be made to appreciate the real character of street dust."

We should be derelict of duty should we fail to hang out a *danger* signal though unheeded, amid the besetting foulness in which fashion of to-day wallows and drags to our several homes.

The N. Y. *Sun*, in an editorial on dress reform, truly says that "until the women of Fifth avenue set the fashion of dress reform, there will be no reform in the feminine costume in any quarter, though nothing is more obvious than the total inappropriateness of the present dress of working women. Distinctions in dress which mark distinctions in employment, are intolerable in the eyes of women."

It is the queens of fashion, the leaders of society, to whom we must look for any substantial reform in matters of dress. What say they?

